# Chit-Chat

**Published:** 1880-10

**Authors:** 


### Editorial Chit-Chat

HARASSING.—To pick up your daily paper after some feminine member of the household has had the first handling of it. A woman can turn a newspaper wrong side out and upside down quicker and get more news out of it while so tangling it, than any other being of equal intelligence and loveliness.

DESIRABLE PREMIUM.—We perceive, from the published premium list of the Chemung County Fair, that a certain young lady was awarded a husband as third premium for the best plate of raised biscuit. As the first premium was two dollars in cash, one wonders what sort of a husband was awarded at so small a valuation.

THAT HOME.—That enthusiastic band of woman who have been heartily engaged—for a year or more—in building a home for the aged have completed their labors and opened their house to the inspection of the public. A right comfortable home they have made of it too, and have commenced housekeeping with one old gentleman and five old ladies. One needs only to call upon these contented, happy old folks to be satisfied that the institution is a commendable one, right worthy of public support.

A NOVEL IDEA.— The excellent lady managers of the "Home for the Aged" in our city are contemplating a rather unusual department for an institution devoted to the care of old men and women. We have it upon the authority of one of the most enthusiastic managers that they are fitting up a "nursery" in that institution. It occurs to us that such a department seems superfluous in view of the fact that the Orphans' Home might be utilized should such a department become really necessary.

REV. E. P. ADAMS' CASE.—This gentleman has been deposed from the ministry of the Presbyterian church at Dunkirk, N. Y., for heresy, in denying the existence of the orthodox everlasting-hell. We made inquiry of a gentleman from that village as to why his attempted expulsion from the church should have been conducted so quietly—without exciting strife and ill-feeling among the members of his congregation. "Because," said the gentleman, "Mr. Adams does not want to go to hell nor have any one else go, and nobody wants him to; while his principal opponent is determined to go there, and everybody is willing he should!"

THOSE TWO SOCIETIES having in charge the Adam monument project, and the cremation furnace, have by no means subsided; they are only resting and awaiting the advent of cold weather when the members of the several committees will renew their efforts to bring both enterprises to a successful consummation.

The monument to Adam will certainly be builded and it is to be hoped a sufficient sum can be raised for a cremation furnace, also.

No punning or commenting upon this paragraph will be entertained.

AN INVENTION that failed because of its success is related of Prof. E. S. Morse, by a friend. When the professor was a young man and too poor to keep a servant, his wife did the cooking, and he was compelled to rise earlier than was his wont, to build the kitchen fire. He soon brought his inventive genius to his aid and constructed an apparatus by which he was able to pull a string suspended over his bed and attached to a match arranged in the combustible material in the stove, which was prepared the night before, and which lighted the fire without the necessity of his getting out of bed.

He declared the devise worked well enough, but lost its value by reason of his invariably getting up to see whether the blamed thing "went off" or not.

ORDER ASKED FOR.—We respectfully, but none the less anxiously, call attention of the janitor of the meeting house where we are in the habit of attending divine service on Sunday, that until he removes that feather duster, mounted upon a fish-pole, and which is allowed to recline against the wall in rear of the pastor, some what gracefully, but singularly poised at an angle of about 95° with the plane of the eliptic, the most excellent sermons there promulgated are likely to be entirely lost upon the subscriber. He will readily perceive that a fellow can not speculate upon the force requisite to precipitate that bunch of turkey feathers upon the ministers' head, and so convert him into an impromptue Comanche chief, or perhaps fall of its own gravity upon the choir and elicit a note from the soprano not laid down in the music, with out somewhat distracting a fellow's attention, thereby losing to him the best points in the discourse. Then, we object to the ornamentation —if it is so intended, because of the absence of anything on the opposite side of the organ to properly carry out the idea of bilateral conformation. Place a sun-flower stalk there—blossom and all, that our ideas of beauty of arrangement may not be offended, and we'll stand it, otherwise we mean to make a fuss about it.

---

THE ELMIRA MICROSCOPICAL SOCIETY.—The last meeting of this enthusiastic body of scientists was an unusually interesting and profitable one. The attendance was large and the evening very profitably spent in viewing and discussing the beautiful creatures of the microscopic world.

Dr. Geo. E. Blackham, the famous microscopist, accepted an invitation to be present and gave the society an illustrated lecture upon wide-angled objectives, detailing their construction and the great advantage derived from their use. The lecture was an exceedingly interesting and instructive one, coming from one who may be said to be the champion if not the father of wide angled objectives.

The society have in contemplation a soiree, to be given in one of our public halls, in which several distinguished microscopists from abroad will aid. Those who receive cards of invitation may consider themselves fortunate, as the number will be limited.

---

LOCOMOTIVE WHISTLES.—Of all the infernal abominations that ever afflicted a patient and long suffering community, the locomotive whistle is the most exasperating and persistent. For so entirely unnecessary an annoyance, it is monstrous. One needs only to reside within a block of the railway, in the Fifth ward of this city, to be daily—almost hourly wrought to a swearing pitch by the demoniacal screech of those three whistles that call for the man at the junction to open the switch to let the screaming devils in. It has been in the past and is now a very serious shock to sick people and a continual and never ceasing disturbance to those who desire to sleep in the night season. We respectfully call the attention of our common council to the matter, asking an abatement of this nuisance; particularly as it will be no inconvenience to the railroad authorities to arrange other signals unaccompanied by any noise.

MISS NEILSON.—Several years ago, when this renowned actress, whose death has been announced, visited this city, she had occasion to call upon a surgeon who tied a varicose vein on her pretty le—limb.

"Do you think this will cause me to limp, to-night?" she inquired.

"Perhaps it may," said the surgeon, "and," he continued, "if it does, I shall certainly receive you with applause."

"In that case, I shall surely limp," she replied.

That night, when she first appeared upon the scene in her great character of Juliet, she purposely made an attempt at a limp, and then looked enquiringly about for her surgeon. She soon discovered his where-abouts in the audience, when he commenced a vigorous clapping of his hands that soon led to tumultuous applause throughout the entire house. To this reception from the audience she bowed graciously and bestowed a bewitching glance upon her physician as much as to say "you did it," and then resumed her role.—The surgeon was the recipient of a beautiful portrait of the handsome actress the following day, and takes pleasure in relating this little incident to those who admire the picture that ornaments his private office.

---

THE CARDINAL FLOWER.—It is very rare here-a-bouts and is very pretty. This makes it desirable; for, the scarcer anything is, the more anxious the average citizen is to possess it, even if it is good for nothing after he gets it. A chap of our acquaintance, recently went in search of the flower named. He went on Sunday as well as on a horse's back, both of which circumstances may account for what happened.

The scarlet blossom was seen on the bank of a brook, in a meadow, near the public road.

The cavalryman instead of dismounting and climbing the fences, jumped his steed over the bars. The horse leaped higher and further than the rider was prepared for, the consequence being a fine bunch of cardinal flowers and a wretchedly lame back in the person of its posessor.

The Sunday ride and bouquet cost the chap spoken of, just two weeks of excruciating suffering in bed, with multitudinous fomentations, cuppings and similar delightful contrivances applied over the region of his back, just where he persisted in declaring his kidneys were located. We simply refer to the matter so that it may be placed on record that Sunday—cardinal flowers—horse-back riding and kidneys, somehow, do not seem to harmonize when placed in one grouping.

---

THE ONLY WAY to make the present medical law a success in accomplishing the banishment of quacks, is to pass another law compelling all the inhabitants of this State to become intelligent and educated human beings.

The reason why ignorant, perambulating quacks thrive, is, that the great majority of people are ignorant and superstitious, and it is upon these that the quack doctors subsist.

---

A COMPANION DIES.—To read a death announcement is in itself rarely startling. Statesmen, warriors, men of letters and of science die ; we read in our daily newspaper the obituary notice of the well known name, shake the ashes from our cigar and seek for a more entertaining theme, without permitting any recollection of the dead man to ruffle our complacency. How rarely are we found dwelling upon the thought that somewhere, the living are suffering anguish—perhaps dispair, by reason of that death. No matter how distinguished the man, how skillful his hand or eloquent his tongue, some one is found to occupy his place, and the great business world moves on as smoothly and undisturbed as before.

But when death's shaft strikes nearer home and carries away a dearly loved friend, then we give time to meditation and to fondly dwelling upon the noble characteristics and lovable traits in him who has greeted us for the last time. Following then, this inclination, we write what we know of him : Were we called upon to pen a single sentance, revealing the character of our friend A. L. Williams,—now dead, we should say "he loved the birds and flowers." Loved them with a consuming, never ceasing ardor, that lighted up his face and sparkled in his tender eye, at the very glimpse of them. Many times have we seen him leave the stream, when engaged in the gentle art, to climb a precipitous bank, there to fondle a delicate anemone which his keen eye had discovered. Seated upon the grassy bank of a rippling mountain stream, we have listened to his eloquent praise of some favorite flower, or of the song of a well known bird. His eye ever on the alert for a new wild flower ; his ear quick to catch the first note of every feathered songster. His acquaintance with Nature extended beyond that gleaned from books—it was a familiar knowledge born of an intimate companionship with the creatures of his choice. Every tiny flower that struggled for an existence in the tangled grass, or beneath a fallen tree found a ready, cheerful rescuer at his hands, for he knew their haunts and searched for them with a never failing delight. To hear him talk to the birds and comment upon their habits, was alone richly worth a day's ramble in his company. The bird that chirped unseen in the branches of a tree or soared away to the clouds, was quickly recognized and some peculiar habit unfolded to the companion who cared to listen. A taxidermist of considerable skill, it was not strange that his dwelling was ornamented with the beautiful creatures that he so much loved. As he pointed out each pretty specimen of his handiwork, a little story of its capture was always related, revealing the great enjoyment that his excursions to the woods invariably engendered. Bird-nests of various kinds hung suspended by the picture frames of his sitting room, at home ; birds were grouped in various attitudes about the apartment, while ferns and wild flowers grew in cones, and from brackets constructed of fungus growths taken from the forest trees. Thus, in his dwelling, was his fondness for nature manifested.

His acquaintance with literature and science was but little less than that of Nature, giving to his conversation that charm inseparable from a well stored mind. Attractive in person, delightfully entertaining of speech, skilled in the use of rod and gun, thoroughly familiar with Nature, he could not be other than a most charming acquaintance. He despised ostentation ; disliked all kinds of display ; forsook demonstrative religion, and took his stand, as upon a solid rock, in this enunciation of his faith : "I believe in one God—the Father Almighty, projector of this fair earth of ours ; and in this my creed—that I shall conduct myself, while here, the very best I know how !"

Williams is dead ! To those who knew him as well as the writer, a deep, everlasting regret must accompany the announcement. Many men die every day, but none more deserving of life hereafter, than he. May the flowers bloom ever, over his peaceful resting-place—the birds sing their choicest melodies there, and may he see and hear them too, as of yore.

